# Rapid Screening of Chemical Constituents in* Rhizoma Anemarrhenae* by UPLC-Q-TOF/MS Combined with Data Postprocessing Techniques

**DOI:** 10.1155/2017/4032820

**Published:** 2017-09-25

**Authors:** Lanlan Shan, Yuanyuan Wu, Lei Yuan, Yani Zhang, Yanyan Xu, Yubo Li

**Affiliations:** Tianjin State Key Laboratory of Modern Chinese Medicine, School of Traditional Chinese Materia Medica, Tianjin University of Traditional Chinese Medicine, 312 Anshan West Road, Tianjin 300193, China

## Abstract

*Rhizoma Anemarrhenae*, a famous traditional Chinese medicine (TCM), is the dried rhizome of* Anemarrhena asphodeloides *Bge. (*Anemarrhena* Bunge of Liliaceae). The medicine presents anti-inflammatory, antipyretic, sedative, and diuretic effects. The chemical constituents of* Rhizoma Anemarrhenae* are complex and diverse, mainly including steroidal saponins, flavonoids, phenylpropanoids, benzophenones, and alkaloids. In this study, UPLC-Q-TOF/MS was used in combination with data postprocessing techniques, including characteristic fragments filter and neutral loss filter, to rapidly classify and identify the five types of substances in* Rhizoma Anemarrhenae*. On the basis of numerous literature reviews and according to the corresponding characteristic fragments produced by different types of compounds in combination with neutral loss filtering, we summarized the fragmentation patterns of the main five types of compounds and successfully screened and identified 32 chemical constituents in* Rhizoma Anemarrhenae*. The components included 18 steroidal saponins, 6 flavonoids, 4 phenylpropanoids, 2 alkaloids, and 2 benzophenones. The method established in this study provided necessary data for the study on the pharmacological effects of* Rhizoma Anemarrhenae* and also provided the basis for the chemical analysis and quality control of TCMs to promote the development of a method for chemical research on TCMs.

## 1. Introduction

*Rhizoma Anemarrhenae* is one of the traditional Chinese medicines (TCMs) that are commonly used for clearing heat and purging fire, nourishing yin, moistening dryness, reducing anxiety and thirst, and promoting excretion. This TCM is the dried rhizome of the Liliaceae* Anemarrhena* genus and has been widely used for clinical treatment of febrile disease, high fever and thirst, cough and asthma, osteopyrexia and fever, and other diseases [1, 2]. The pharmacologically active components of* Rhizoma Anemarrhenae* include steroidal saponins, flavonoids, phenylpropanoids, alkaloids, and benzophenones, with saponins being the major constituents [3–5]. At present, the analytical methods of TCM ingredients mainly include thin layer chromatography (TLC), gas chromatography (GC), high-performance liquid chromatography (HPLC), and ultraperformance liquid chromatography/time-of-flight mass spectrometry (UPLC-Q-TOF/MS), among others. As a comprehensive analysis technique, UPLC-Q-TOF/MS has been increasingly applied for the qualitative and quantitative analysis of components in TCMs and compound preparations [6–11]. However, this method displays several disadvantages, including complicated and time-consuming data processing. Therefore, a newly developed method to rapidly identify components in TCMs is urgently needed to improve the efficiency and accuracy of the qualitative analysis and to promote the development of modernization and internationalization of TCMs.

With the rapid development of modern technology, data postprocessing techniques, mainly including characteristic fragments filter (CFF) and neutral loss filter (NLF), play increasingly important roles in TCM research [12–17]. CFF is based on compounds with the same or similar mother nucleus structures that can produce characteristic fragments to determine a certain type of compound under the same mass spectrometric conditions. NLF is based on a certain type of compounds that frequently lose the same neutral fragments by energy collision splitting. According to the loss of these neutral fragments, substances with a class of characteristic substituted groups can be briefly classified [18–22]. CFF is favorable for the rapid screening of similar compounds, whereas NLF helps further confirmation of compounds. Thus, the combination of both techniques markedly reduces qualitative analysis difficulty and was gradually applied in the field of pharmaceutical analysis.

In recent years, UPLC-Q-TOF/MS is widely used to analyze constituents in* Rhizoma Anemarrhenae* [23, 24]. In this study,* Rhizoma Anemarrhenae* was selected as an example to establish an efficient approach for the rapid screening of chemical components by using UPLC-Q-TOF/MS in combination with data postprocessing techniques. First, through reading and integration of abundant information from the literature, we summarized the fragmentation patterns of five kinds of compounds (steroidal saponins, flavonoids, phenylpropanoids, alkaloids, and benzophenones) in* Rhizoma Anemarrhenae*. Secondly, a full spectrum scan was performed by UPLC-Q-TOF/MS. Finally, the compounds were characterized based on the CFF and NLF rule. Compared with traditional methods, the study could rapidly classify and identify a certain type of compounds. The method of UPLC-Q-TOF/MS coupled with data postprocessing techniques not only partly overcame the difficulty of rapid classification and identification of complex components of TCM but also laid the foundation for the rapid development of composition identification and provided an analytical method for the further research and development of TCMs.

## 2. Experimental

### 2.1. Sample Preparation

Prepared* Rhizoma Anemarrhenae* was accurately weighed (5 g) and extracted twice with 50 and 40 mL of 75% methanol, respectively, and each reflux time was 1 h. The extracts were filtered, merged, and then concentrated to 0.01 g/mL. The concentrated solution was filtered using a 0.22 *μ*m syringe filter, with 2 *μ*L injected for UPLC-Q-TOF/MS analysis.

### 2.2. UPLC-Q-TOF/MS Conditions

Chromatographic separation was conducted on a Waters Acquity UPLC Class I series, which consisted of a quatpump, an autosampler, a DAD detector, and a column compartment. All separation was carried out on a Waters ACQUITY UPLC BEH C_18_ column (100 mm × 2.1 mm, 1.7 *μ*m particle size) with a set column temperature of 35°C. The solvent flow rate was 0.3 mL/min, and 2 *μ*L of sample solution was injected in each run. The mobile phase was composed of water (solvent A) and acetonitrile (solvent B). The gradient elution program employed was as follows: 0–0.5 min, 2%–5% B; 0.5–3 min, 5%–20% B; 3–5 min, 20%–22% B; 5-6 min, 22%-22% B; 6–10 min, 22%–38% B; 10–14 min, 38%–50% B; 14-15 min, 50%-50% B; 15–18 min, 50%–100% B; 18–20 min, 100%-100% B; 20-21 min, 100%–2% B; 21–23 min, 2%-2% B.

UPLC was coupled to a Q-TOF-MS system (Waters, USA) equipped with an electrospray ionization source (ESI) for scanning samples in negative ion modes. Ultrahigh purity helium (He) was used as the collision gas, and high-purity nitrogen (N_2_) was used as nebulizing gas. The conditions of ESI source were as follows: drying gas temperature: 325°C; drying gas flow rate: 10 mL min^−1^; desolvation gas flow: 600 L/h; capillary voltage: 2.0 kV; collision energy: 20–40 eV; scan spectra from* m/z* 50–1500; nebulizing gas pressure: 350 psi. Leu-enkephalin ions at* m/z* 556.2771 and 554.2615 were used to ensure accuracy in spectral acquisition.

### 2.3. Data Analysis

Original data were analyzed using Masslynx (Waters, USA) software 4.1. to detect and align the peaks. Data were processed and converted to an Excel format containing complete information on mass, retention time, and peak area of the samples. Target compounds were obtained by output data processing.

## 3. Results and Discussion

### 3.1. Method Development

The chemical constituents of* Rhizoma Anemarrhenae* are complex and diverse, but similar kinds of compounds exhibit certain similarities in chemical structures. We summarized the fragmentation patterns of compounds in* Rhizoma Anemarrhenae* according to the mass spectrometric behavior; that is, the same type of compounds with the same or similar public skeleton usually show the same fracture mode to produce the same CFs. The method that uses CFs to determine a certain type of compounds is called CFF. For example, phenylpropanoid compounds can produce characteristic fragment ions at* m/z *107 [C_7_H_7_O]^−^ and 91 [C_7_H_7_]^−^ in negative ion mode. The ions could be screened from the total ion current chromatograms according to their CFs and then identified according to specific fragments and molecular ion peaks. In MS collision-induced dissociation, the differences between molecular ion peaks and high mass-to-charge ratio fragment peaks also play an important role in component identification. The method that uses NLs to screen compounds is called NLF. Mangiferin could easily lose neutral fragments at* m/z* 90 (C_3_H_6_O_3_) and 120 (C_4_H_8_O_4_) and could be filtered according to the two characteristic neutral fragments. CFF and NLF perform an increasingly important role in TCM research. Therefore, the complex components in* Rhizoma Anemarrhenae* can be rapidly and accurately classified and identified by combining UPLC-Q-TOF/MS with data postprocessing techniques (CFs and NLs). Finally, Based on the results of our study and those described in relevant and previous experiments, the rules of CFF and NLF regarding the five categories were established (shown in Table 1).

**Figure 1:**
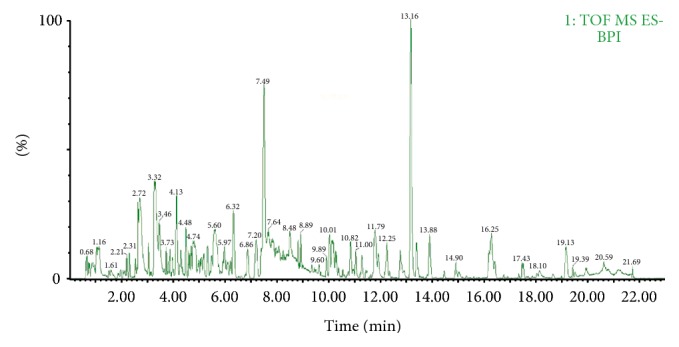
Base peak ion (BPI) chromatograms of substances in the extract of the* Rhizoma Anemarrhenae* herb, under negative ion mode.

A full scan was conducted in both positive and negative modes to analyze the* Rhizoma Anemarrhenae* extract, and results show that the ESI negative ion mode was simpler, more stable, and easier to interpret compared with the positive ion mode, and the characteristic ions obtained in the negative ion mode could be evidently observed to distinguish the two types of steroidal saponins. Thus, the negative ion mode was used in this study. The full scan MS chromatograms of substances in* Rhizoma Anemarrhenae* extracts are shown in Figure 1. The fragmentation patterns of the five types of main constituents in* Rhizoma Anemarrhenae* were summarized, and 32 compounds were identified (shown in Table 2).

### 3.2. Steroidal Saponins

Certain cleavage law exists under a certain MS condition for steroidal saponins. Steroidal saponins can be divided into two types: furostanol glycosides, which bear an open side chain at C-22, and spirostanol glycosides, which contain a closed spiroketal ring at C-22. Based on abundant fragment ions generated by losing sugar chains, side chains and dehydration steroidal saponins were divided into types I to V according to the numbers of hydroxyl group and double bond in the A, B, C, and D rings [23, 24, 27, 30].

First, [M-H-OH]^−^ or [M-H-OCH_3_]^−^ molecular ion peaks were obtained as the CFs of furostan saponins, which display C22-OH or C22-OCH_3_ on their structures, because furostan saponins preferentially lose the hydroxyl or methoxy on C-22. In contrast, spirostanol saponins generally produced [M-H]^−^ molecular ion peaks. In the negative ion mode, type I saponins produce the same fragment ions at* m/z *273 [C_19_H_29_O]^−^, 255 [C_19_H_27_]^−^, 301 [C_21_H_33_O]^−^, and 283 [C_21_H_31_]^−^; types II to IV produce the same fragment ions at* m/z* 271 [C_19_H_27_O]^−^, 253 [C_19_H_25_]^−^, 299 [C_21_H_31_O]^−^, and 281 [C_21_H_29_]^−^; and type V saponins produce the same fragment ions at* m/z* 269 [C_19_H_25_O]^−^ and 251 [C_19_H_23_]^−^. Type IV saponins could be determined when fragment ions at* m/z* 271 [C_19_H_27_O]^−^, 253 [C_19_H_25_]^−^, 299 [C_21_H_31_O]^−^, and 281 [C_21_H_29_]^−^ are generated, whereas type II and type III saponins can be identified when fragment ions at* m/z* 289 [C_19_H_29_O_2_]^−^ and 317 [C_21_H_33_O_2_]^−^ appear in addition to the above-mentioned fragments. Fragment ions at* m/z* 271 [C_19_H_27_O]^−^ and 299 [C_21_H_31_O]^−^ resulted from the loss of sugar chains and side chains, followed by further loss of one water molecule for type II and type III saponins and the loss of the sugar chains and side chains only for type IV saponins. All steroidal saponins produce abundant characteristic fragment ions through the loss of the branched glycoside chains and side chains or by dehydration; the ions could be rapidly identified by combining CFs with NLs at* m/z* 162 (C_6_H_10_O_5_), 132 (C_5_H_8_O_4_), 142 (C_8_H_14_O_2_) and 18 (H_2_O). We used compounds purpureagitoside, timosaponin H1, and timosaponin AIII to discuss the fragmentation pathways in detail in the successive ESI–MS^*n*^ experiments [5, 23, 24, 27, 30].

**Figure 2:**
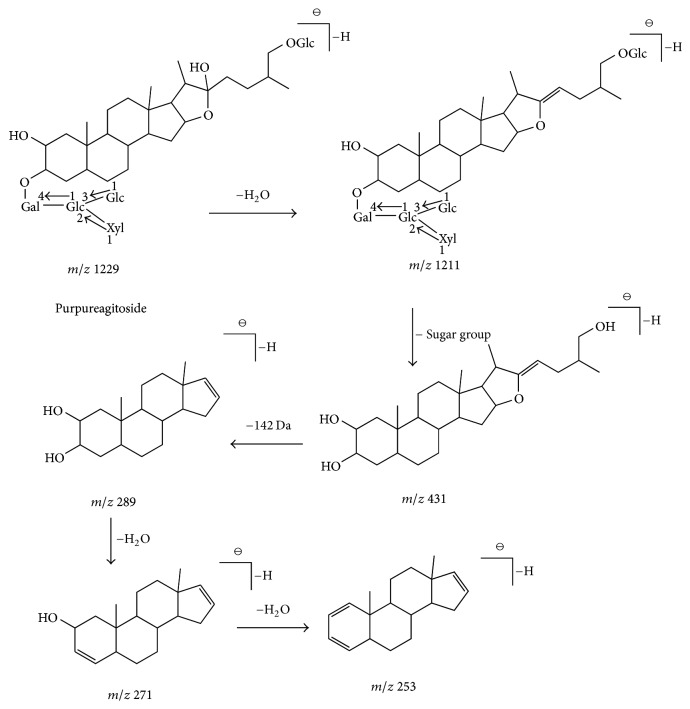
The fragmentation patterns of furostan saponins (type III), taking purpureagitoside as the example: the ion at* m/z* 1229 was the molecular ion [M-H]^−^, the fragmentation at* m/z* 1211 indicated a loss of 18 Da (H_2_O) on the basis of the parent ion, the fragment ions* m/z* 431 displayed the loss of sugar group, the fragment ion (*m/z* 289) was formed by the cleavage of the E-ring on the basis of the fragment ion (*m/z* 431), and the fragment ions (*m/z* 271 and 253) were obtained by a loss of 18 Da (H_2_O) or 2 × 18 Da (H_2_O) on the basis of the fragment ion (*m/z* 289).

Compound 8 presented a retention time of 5.09 min and formula of C_56_H_94_O_29_. Six fragment ions at* m/z* 1229 [M-H]^−^, 1211 [M-H-H_2_O]^−^, 289 [M-H-H_2_O-C_5_H_8_O_4_-4C_6_H_10_O_5_-C_8_H_14_O_2_]^−^, 271 [M-H-2H_2_O-C_5_H_8_O_4_-4C_6_H_10_O_5_-C_8_H_14_O_2_]^−^, 253 [M-H-3H_2_O-C_5_H_8_O_4_-4C_6_H_10_O_5_-C_8_H_14_O_2_]^−^, and 281 [M-H-3H_2_O-C_5_H_8_O_4_-4C_6_H_10_O_5_-C_6_H_10_O_2_]^−^ were detected in negative ion mode. The compound exhibit a molecular ion peak 1229 [M-H]^−^. The main fragment ions at* m/z* 289, 271, 253, and 281 were generated from the losses of sugar chain, along with the cleavage of the E-ring and dehydration. The fragment ion at* m/z* 289 displayed the sequential losses of 132 Da for a xyl molecule, 4 × 162 Da for four hexose molecules, and 142 Da (C_8_H_14_O_2_) based on the fragment ion at* m/z* 1211 [M-H-H_2_O]^−^. The fragment ion at* m/z *271 [M-H-2H_2_O-C_5_H_8_O_4_-4C_6_H_10_O_5_-C_8_H_14_O_2_]^−^ was derived from the loss of one water molecule on the basis of the losses of sugar chains and side chains. According to NLs rules, Compound 8 can be inferred as furostanol glycosides. The fragment ion at* m/z* 1211 [M-H-H_2_O]^−^ indicated the presence of hydroxyl at the position of C-22 due to the fact that furostanol glycosides preferentially lose hydroxyl located in C-22. Compound 8 can be identified as purpureagitoside through comparison with the literature [5, 27]. The fragmentation patterns of purpureagitoside are presented in Figure 2.

Compound 13, with a retention time of 8.12 min and a formula of C_56_H_92_O_28_, exhibited fragment ions at* m/z* 1211 [M-H]^−^, 1193 [M-H-H_2_O]^−^, 271 [M-H-H_2_O-C_5_H_8_O_4_-4C_6_H_10_O_5_-C_8_H_14_O_2_]^−^, and 253 [M-H-2H_2_O-C_5_H_8_O_4_-4C_6_H_10_O_5_-C_8_H_14_O_2_]^−^ in the MS spectrum. The compound produced molecular ions at* m/z* 1211 [M-H]^−^. The main fragment ions at* m/z* 271 [M-H-H_2_O-C_5_H_8_O_4_-4C_6_H_10_O_5_-C_8_H_14_O_2_]^−^ and 253 [M-H-2H_2_O-C_5_H_8_O_4_-4C_6_H_10_O_5_-C_8_H_14_O_2_]^−^ were both derived from the losses of sugar chain, 142 Da (C_8_H_14_O_2_), and water molecule in the MS^2^ spectrum, whereas the fragment ion of* m/z* 289 was not detected according to CFs and NLs rules shown in Table 1. Compound 13 was revealed as a furostanol glycoside (type IV). The fragment ion at* m/z* 1193 [M-H-H_2_O]^−^ suggests that one hydroxyl group exists at the C-22 position of the compound because furostanol glycosides preferentially lose hydroxyl in C-22. The fragment ion at* m/z* 271 [M-H-H_2_O-C_5_H_8_O_4_-4C_6_H_10_O_5_-C_8_H_14_O_2_]^−^ was produced by the losses of sugar chains and side chains only. Thus, compound 13 was identified as timosaponin H1 [5, 27].

**Figure 3:**
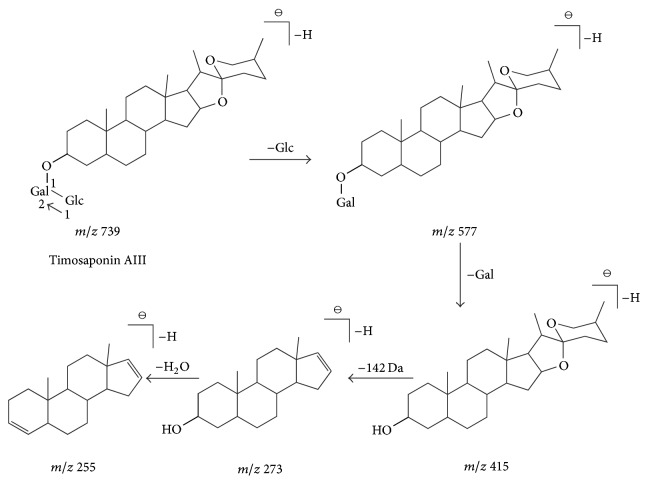
The fragmentation pathways of spirostanol saponins, taking purpureagitoside as the example: the ion at* m/z* 739 was the molecular ion [M-H]^−^, the ion at* m/z* 577 [M-H-C_6_H_10_O_5_]^−^ was produced by the loss of a hexose unit (Glc, 162 Da), the fragment ion at* m/z* 415 [M-H-2C_6_H_10_O_5_]^−^ could be explained by ejecting a pentose unit (Gal, 162 Da) from the ion at* m/z* 577, the fragment ion at* m/z* 273 was formed by the cleavage of the E-ring, and the fragmentation of* m/z* 255 was obtained by the fragmentation* m/z* 273 to lose a water molecule.

Compound 29 presented a retention time of 16.17 min and a formula of C_39_H_64_O_13_. Several main fragment ions at* m/z* 739 [M-H]^−^, 577 [M-H-C_6_H_10_O_5_]^−^, 415 [M-H-2C_6_H_10_O_5_]^−^, 273 [M-H-2C_6_H_10_O_5_-C_8_H_14_O_2_]^−^, and 255 [M-H-2C_6_H_10_O_5_-C_8_H_14_O_2_-H_2_O]^−^ were obtained in the mass spectrum. The compound was judged to be type I saponins according to the CFs of* m/z* 273 [M-H-2C_6_H_10_O_5_-C_8_H_14_O_2_]^−^ and 255 [M-H-2C_6_H_10_O_5_-C_8_H_14_O_2_-H_2_O]^−^. The ion at* m/z* 577 [M-H-C_6_H_10_O_5_]^−^ was produced by the loss of a hexose unit (Glc, 162 Da) from the molecular ion at* m/z *739 [M-H]^−^. The fragment ion at* m/z* 415 [M-H-2C_6_H_10_O_5_]^−^ could be explained by the ejection of a hexose unit (Gal, 162 Da) from the ion at* m/z* 577. The mass-to-charge ratio difference between the two fragment ions (*m/z* 415 and 273) was 142 Da, thereby suggesting that the fragment ion at* m/z* 273 was formed by the cleavage of the E-ring. Fragmentation at* m/z* 255 was obtained by the fragment ion* m/z* 273 to lose a molecule H_2_O. According to CFs and NLs rules shown in Table 1, compound 29 was revealed as spirostanol saponins (type I). These results are consistent with the fragmentation pathway of timosaponin AIII [5, 27]. A proposed mechanistic pathway for fragments formed in MS is shown in Figure 3.

### 3.3. Flavonoids

Flavonoids were divided into mangiferin-type flavonoids, chalcone-type flavonoids, flavanones, homoisoflavonoids, and icariin-type flavonoids according to their chemical structures. Mangiferin-type flavonoids shared the same aglycone structure, the aglycone and sugar of which formed C-glycosides, and C-glycosides tended to lose CH_2_O units during MS, and 2/3 and 3/4 CH_2_O units could be eliminated from sugar molecules through ^0,3^X/^0,2^X cleavage [5, 25]; therefore, under the negative ion mode, the C-glycosides easily lost the neutral fragments at* m/z* 90 (C_3_H_6_O_3_) and 120 (C_4_H_8_O_4_), enabling the rapid and efficient identification of mangiferin-type flavonoids based on the two characteristic neutral loss ions. The structures of chalcone-type flavonoids possessed the same B ring and C ring cracking, with fragment ions at* m/z* 119 [C_8_H_7_O]^−^ or 146 [C_9_H_6_O_2_]^−^ produced by A and B ring cleavage during MS (negative ion mode). The compounds can be screened depending on these two characteristic fragment ions. Homoisoflavonoids exhibit the same B ring structure and reverse Diels-–Alder (RDA) of the C ring mainly occurred, producing fragment ion at* m/z* 133 [C_9_H_9_O]^−^, which could be recognized as the characteristic fragment ion of these compounds. Flavanones formed two main complementary fragments at* m/z* 119 [C_8_H_7_O]^−^ and 165 [C_8_H_5_O_4_]^−^ by C ring for RDA cleavage. Anthocyanin aglycones possess the same aglycone structure and could be rapidly identified according to their aglycone fragment of* m/z* 367 [C_21_H_19_O_6_]^−^ and neutral loss molecules of* m/z* 162 (C_6_H_10_O_5_) and 132 (C_5_H_8_O_4_) [5, 25, 29, 31, 32].

**Figure 4:**
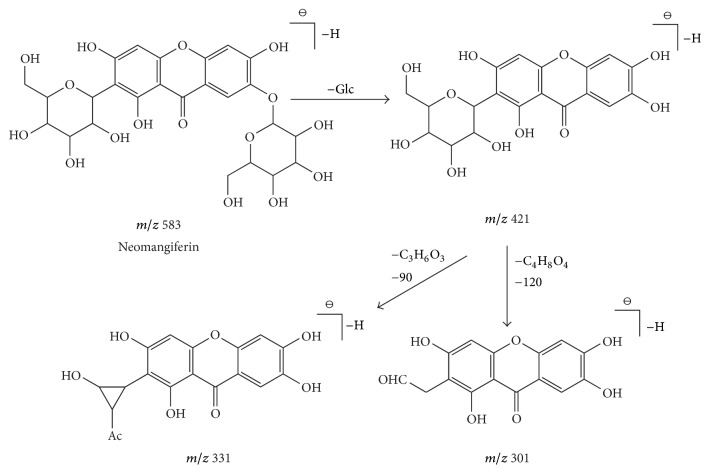
The specific fragmentation process of mangiferin flavonoids, take neomangiferin as an example: The fragment ion at* m/z* 583 [M-H]^−^ was a molecular ion,* m/z* 421 displayed a loss of 162 Da (C_6_H_10_O_5_) based on the parent ion, the fragment ion at* m/z* 331 [M-H-C_6_H_10_O_5_-C_3_H_6_O_3_]^−^ conformed to the rule of C-glycosides loss 90 Da (C_3_H_6_O_3_) and* m/z* 301 [M-H-C_6_H_10_O_5_-C_4_H_8_O_4_]^−^ followed the rules of C-glycosides loss 120 Da (C_4_H_8_O_4_) from sugar molecules through ^0,3^X/^0,2^X cleavage.

Compound 1 presented a retention time of 3.55 min and a formula of C_25_H_28_O_16_, generating product ions at* m/z* 583 [M-H]^−^, 421 [M-H-C_6_H_10_O_5_]^−^, 331 [M-H-C_6_H_10_O_5_-C_3_H_6_O_3_]^−^, and 301 [M-H-C_6_H_10_O_5_-C_4_H_8_O_4_]^−^. The fragment ion at* m/z* 583 [M-H]^−^ was a molecular ion. In addition, we obtained fragment ions at* m/z* 421 [M-H-C_6_H_10_O_5_]^−^ that displayed a loss of 162 Da [C_6_H_10_O_5_] based on the parent ion. We also obtained fragment ions at* m/z* 331 [M-H-C_6_H_10_O_5_-C_3_H_6_O_3_]^−^ and 301 [M-H-C_6_H_10_O_5_-C_4_H_8_O_4_]^−^, which corroborated with the rules of C-glycoside loss of 90 (C_3_H_6_O_3_) and 120 Da (C_4_H_8_O_4_) from sugar molecules through ^0,3^X/^0,2^X cleavage. These results follow the rules of CFs and NLs shown in Table 1. Compound 1 was rapidly and efficiently revealed as mangiferin-type flavonoids. Therefore, compound 1 was determined to be neomangiferin [5, 25]. The specific fragmentation process of neomangiferin is shown in Figure 4.

**Figure 5:**
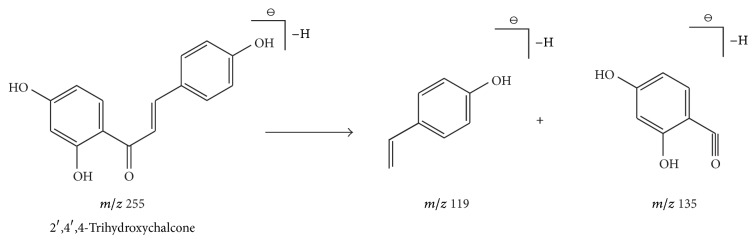
The specific fragmentation process of chalcone flavonoids, take 2′,4′,4-trihydroxychalcone as an example: the fragment ion of* m/z* 255 [M-H]^−^ is molecular ion and the fragment ions at* m/z* 135 and 119 resulting from separation of A and B rings.

Compound 22 possessed a retention time of 10.69 min and a formula of C_15_H_12_O_4_. In the negative ion mode, several fragment ions at* m/z* 255 [M-H]^−^, 135 [M-H-C_7_H_4_O_2_]^−^, and 119 [M-H-C_7_H_4_O_3_]^−^ were detected; among the fragment ions, those at* m/z* 135 [M-H-C_7_H_4_O_2_]^−^ and 119 [M-H-C_7_H_4_O_3_]^−^ resulted from the cleavage by separation of A and B rings, and the fragment ion at* m/z* 119 [M-H-C_7_H_4_O_3_]^−^ corresponded to the typical fragment of chalcone-type flavonoids according to CFs and NLs rules shown in Table 1. Besides, the fragment ion at* m/z* 255 [M-H]^−^ is a molecular ion. Compound 22 was rapidly revealed as chalcone flavonoids. Finally, compound 22 was identified as 2′,4′,4-trihydroxychalcone [29]. The specific fragmentation process of 2′,4′,4-trihydroxychalcone is shown in Figure 5.

### 3.4. Phenylpropanoids

Phenylpropanoid compounds can be classified into lignans and coumarins according to different parent structures. Lignan compounds with the same A ring structure and benzyl groups usually produce the characteristic fragments of* m/z* 107 [C_7_H_7_O]^−^ and 91 [C_7_H_7_]^−^ by benzyl cleavage, as well as other fragment ions because of different linking groups to the A ring; then, the compound was determined according to the molecular ion fragments [29, 33].

Compound 27 presented a retention time of 12.25 min and a formula of C_16_H_18_O_3_. In the negative ion mode, several fragment ions of* m/z* 257 [M-H]^−^, 107 [M-H-C_9_H_10_O_2_]^−^, and 91 [M-H-C_9_H_10_O_3_]^−^ were observed; among the fragments, the fragment ion at* m/z* 107 [M-H-C_9_H_10_O_2_]^−^ was produced as a result of benzyl cleavage, and the fragment ion at* m/z* 91 [M-H-C_9_H_10_O_3_]^−^ indicated a loss of water molecule from the fragment ion at* m/z* 107 [M-H-C_9_H_10_O_2_]^−^. These two fragments show that this compound belongs to coumarin according to CFs rules shown in Table 1. Besides, the fragment ion of* m/z* 257 [M-H]^−^ as molecular ion was detected; thus, compound 27 was determined as Broussonin A or Broussonin B [29].

**Figure 6:**
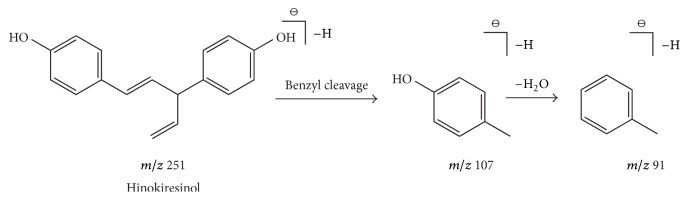
A fragmentation pathway of lignans formed in MS, take hinokiresinol as an example: the fragment ion of* m/z* 251 [M-H]^−^ was molecular ion and the fragment ion at* m/z* 107 was produced as result of benzyl cleavage. The fragment ions* m/z* indicated a loss of water molecular based on the fragment ion at* m/z* 107.

Compound 28 possessed a retention time of 13.16 min and a formula of C_17_H_16_O_2_. In the negative ion mode, several fragments were observed at* m/z* 251 [M-H]^−^, 107 [M-H-C_10_H_8_O]^−^ and 91 [M-H-C_10_H_8_O_2_]^−^ were observed. The fragment ion at* m/z* 107 [M-H-C_9_H_10_O_2_]^−^ was produced as a result of benzyl cleavage. The fragment ions at* m/z* 91 [M-H-C_9_H_10_O_3_]^−^ indicated a loss of water molecule based on the fragment ion at* m/z* 107 [M-H-C_9_H_10_O_2_]^−^. Besides, the fragment ion at* m/z* 251 [M-H]^−^ as a molecular ion was detected. According to CFs rules shown in Table 1, compound 28 shared the same fragmentation pathways with compound 27 and belonged to coumarin. Therefore, compound 28 was determined as hinokiresinol [29, 33]. A pathway for fragments formed during MS is shown in Figure 6.

### 3.5. Benzophenones

Benzophenone compounds possess the same A ring structure, which easily loses 94 Da (A ring) and 44 Da (CO_2_) in the negative ion mode. Therefore, benzophenone compounds in* Rhizoma Anemarrhenae* could be rapidly screened and identified according to the characteristics neutral loss of 94 Da and 44 Da in combination with molecular ion [26, 34].

**Figure 7:**
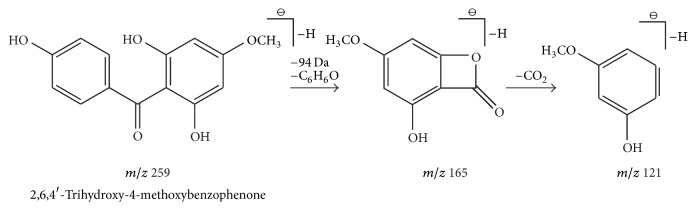
A fragmentation pathway of benzophenone formed in MS, take 2,6,4′-trihydroxy-4-methoxybenzophenone as an example:* m/z* 259 [M-H]^−^ is parent ion, the fragment ion at* m/z* 165 indicated a loss of 94 Da (C_6_H_6_O) based on the parent ion, and the fragment ion at* m/z* 121 indicated a loss of one CO_2_ molecule based on the fragment at* m/z *165.

Compound 12 presented a retention time of 7.49 min and a formula of C_14_H_12_O_5_, thereby showing several fragments at* m/z* 259 [M-H]^−^, 165 [M-H-C_6_H_6_O]^−^, and 121 [M-H-C_6_H_6_O-CO_2_]. The fragment ion at* m/z* 165 [M-H-C_6_H_6_O]^−^ indicates a loss of 94 Da (C_6_H_6_O); the difference between fragment ions (*m/z* 165 and 121) is exactly 44 Da, indicating a loss of one CO_2_ molecule. The behavior follows the NLs rules shown in Table 1, and the compound was identified as benzophenone. Besides,* m/z* 259 [M-H]^−^ is the parent ion; thus, compound 12 was determined as 2,6,4′-trihydroxy-4-methoxybenzophenone [26, 34]. A pathway for fragments formed in MS is shown in Figure 7.

### 3.6. Alkaloids

Alkaloids were divided into amide alkaloids with a N-C=O structure and pyridine alkaloids with N atoms in a six-member ring. In the negative ion mode, amide alkaloid with the same part of the structure easily loses a tyramine residue, resulting in neutral loss of molecule 119 (C_8_H_7_O). In addition, 15 Da (CH_3_) can be easily lost based on the parent ion in the high mass-to-charge ratio. Given the same pyridine ring structure of pyridine alkaloids, the characteristic fragment ion at* m/z* 78 [C_5_H_4_N]^−^ was produced by removing the side chain to form the pyridine ring structure, and compounds were further identified through molecular ions [28, 35].

Compound 10 presented a retention time of 6.86 min and a formula of C_18_H_19_NO_4_ and exhibited several fragments at* m/z* 312 [M-H]^−^, 297 [M-H-CH_3_]^−^, and 178 [M-H-CH_3_-C_8_H_7_O]^−^. The fragment ion at* m/z* 297 [M-CH_3_]^−^ was observed in the high mass-to-charge ratio, displaying a loss of 15 Da (CH_3_) based on the parent ion. In addition, the fragment ion at* m/z* 178 [M-H-CH_3_-C_8_H_7_O]^−^ corroborated with the loss of 119 Da (C_8_H_7_O) based on the fragment ion at* m/z* 297. The behavior followed the CFs and NLs rules shown in Table 1, In addition, the fragment ion at* m/z* 312 [M-H]^−^ was the molecular ion. Thus, compound 10 was identified as* N-trans*-feruloyltyramine or* N-cis*-feruloyltyramine [28].

## 4. Conclusions

Based on the fragmentation patterns of five kinds of compounds in* Rhizoma Anemarrhenae* (as summarized from the literature), a combination of UPLC-Q-TOF/MS with data postprocessing techniques (CFF and NLF) was developed in this study to rapidly classify and identify 32 compounds in* Rhizoma Anemarrhenae* extracts. The results provide a basis for the pharmacological and pharmacokinetic studies on* Rhizoma Anemarrhenae*, overcome the shortage for traditional chemical analysis of TCM to a certain extent, and facilitate the advancement and development of TCM study.

## Figures and Tables

**Table 1:** Characteristic fragments and neutral loss information of chemical substances in the *Rhizoma Anemarrhenae.*

Compound classification	Subclass	Characteristic fragments	Neutral loss
Steroidal saponins			
Spirostanol saponins [M-H]^−^	Type I saponins	273 [C_19_H_29_O]^−^, 255 [C_19_H_27_]^−^, 301 [C_21_H_33_O]^−^, 283 [C_21_H_31_]^−^	162 (C_6_H_10_O_5_), 132 (C_5_H_8_O_4_) 18 (H_2_O) 142 (C_8_H_14_O_2_)
	Type II and type III saponins	289 [C_19_H_29_O_2_]^−^, 317 [C_21_H_33_O_2_]^−^	
	Type IV saponins	271 [C_19_H_27_O]^−^, 253 [C_19_H_25_]^−^, 299 [C_21_H_31_O]^−^, 281 [C_21_H_29_]^−^	
Furostan saponins [M-H-OH]^−^ [M-H-OCH_3_]^−^	Type V saponins	269 [C_19_H_25_O]^−^, 251 [C_19_H_23_]^−^	
Flavonoids	Mangiferin-type flavonoids		90 (C_3_H_6_O_3_), 120 (C_4_H_8_O_4_)
	Chalcone-type flavonoids	119 [C_8_H_7_O]^−^ or 146 [C_9_H_6_O_2_]^−^	
	Flavanones	119 [C_8_H_7_O]^−^, 165 [C_8_H_5_O_4_]^−^	
	Homoisoflavonoid	133 [C_9_H_9_O]^−^	
	Icariin-type flavonoids	367 [C_21_H_19_O_6_]^−^	162 (C_6_H_10_O_5_), 132 (C_5_H_8_O_4_)
Phenylpropanoids	coumarins	107 [C_7_H_7_O]^−^, 91 [C_7_H_7_]^−^	
Benzophenones			94 (C_6_H_6_O) 44 (CO_2_)
Alkaloids	Amide alkaloids		119 (C_8_H_7_O) 15 (CH_3_)

**Table 2:** Identification of the chemical constituents of *Rhizoma Anemarrhenae* extract by using UPLC-Q-TOF/MS in negative ion mode.

	RT	Formula	[M-H]^−^	Experimental *m/z*	ppm	Fragment ions	Chemical name	Chemical types	Ref
1	2.72	C_25_H_28_O_16_	583.1299	583.1299	0.00	583 [M-H]^−^, 421 [M-H-C_6_H_10_O_5_]^−^, 331 [M-H-C_6_H_10_O_5_-C_3_H_6_O_3_]^−^, 301 [M-H-C_6_H_10_O_5_-C_4_H_8_O_4_]^−^,	Neomangiferin	Mangiferin-type flavonoids	[5, 25]
2	3.27	C_19_H_18_O_11_	421.0771	421.0759	2.85	421 [M-H]^−^, 331 [M-H-C_3_H_6_O_3_]^−^, 301 [M-H-C_4_H_8_O_4_]^−^,	Mangiferin	Mangiferin-type flavonoids	[5, 25]
3	3.32	C_19_H_18_O_11_	421.0771	421.0764	1.66	421 [M-H]^−^, 331 [M-H-C_3_H_6_O_3_]^−^, 301 [M-H-C_4_H_8_O_4_]^−^,	Isomangiferin	Mangiferin-type flavonoids	[5, 25]
4	4.48	C_13_H_10_O_5_	245.0450	245.0454	1.63	245 [M-H]^−^, 151 [M-H-C_6_H_6_O]^−^, 107 [M-H-C_6_H_6_O-CO_2_]^−^,	Iriflophenone	Benzophenones	[26]
5	4.74	C_45_H_76_O_20_	935.4852	935.4823	3.10	917 [M-H-H_2_O]^−^, 773 [M-H-C_6_H_10_O_5_]^−^, 289 [M-H-H_2_O-3C_6_H_10_O_5_-C_8_H_14_O_2_]^−^, 271 [M-H-2H_2_O-3C_6_H_10_O_5_-C_8_H_14_O_2_]^−^, 253 [M-H-3H_2_O-3C_6_H_10_O_5_-C_8_H_14_O_2_]^−^, 281 [M-H-3H_2_O-3C_6_H_10_O_5_-C_6_H_10_O_2_]^−^,	Timosaponin E1	Furostanol glycosides	[5, 27]
6	4.79	C_45_H_76_O_20_	935.4852	935.4827	2.67	935 [M-H]^−^, 773 [M-H-C_6_H_10_O_5_]^−^, 289 [M-H-H_2_O-3C_6_H_10_O_5_-C_8_H_14_O_2_]^−^, 271 [M-H-2H_2_O-3C_6_H_10_O_5_-C_8_H_14_O_2_]^−^, 253 [M-H-3H_2_O-3C_6_H_10_O_5_-C_8_H_14_O_2_]^−^,	Timosaponin E	Furostanol glycosides	[5, 27]
7	4.85	C_45_H_76_O_20_	935.4852	935.4836	1.71	935 [M-H]^−^, 773 [M-H-C_6_H_10_O_5_]^−^, 289 [M-H-H_2_O-3C_6_H_10_O_5_-C_8_H_14_O_2_]^−^,	Timosaponin N	Furostanol glycosides	[5, 27]
8	5.09	C_56_H_94_O_29_	1229.5803	1229.5806	0.24	1211 [M-H-H_2_O]^−^, 289 [M-H-H_2_O-C_5_H_8_O_4_-4C_6_H_10_O_5_-C_8_H_14_O_2_]^−^, 271 [M-H-2H_2_O-C_5_H_8_O_4_-4C_6_H_10_O_5_-C_8_H_14_O_2_]^−^, 253 [M-H-3H_2_O-C_5_H_8_O_4_-4C_6_H_10_O_5_-C_8_H_14_O_2_]^−^, 281 [M-H-3H_2_O-C_5_H_8_O_4_-4C_6_H_10_O_5_-C_6_H_10_O_2_]^−^,	Purpureagitoside	Furostanol glycosides	[5, 27]
9	6.32	C_17_H_17_NO_3_	282.1130	282.1132	0.71	282 [M-H]^−^, 163 [M-H-C_8_H_7_O]^−^,	Coumaroyltyramine	Amide alkaloids	[28]
10	6.86	C_18_H_19_NO_4_	312.1236	312.1237	0.32	312 [M-H]^−^, 297 [M-H-CH_3_]^−^, 178 [M-H-CH_3_-C_8_H_7_O]^−^,	N-trans-feruloyltyrami-ne or N-cis-feruloyltyramine	Amide alkaloids	[28]
11	7.20	C_45_H_74_O_19_	917.4746	917.4751	0.54	755 [M-H-C_6_H_10_O_5_]^−^, 289 [M-H-3C_6_H_10_O_5_-C_8_H_14_O_2_]^−^, 271 [M-H-3C_6_H_10_O_5_-C_8_H_14_O_2_-H_2_O]^−^, 253 [M-H-3C_6_H_10_O_5_-C_8_H_14_O_2_-2H_2_O]^−^ 281 [M-H-3C_6_H_10_O_5_-C_6_H_10_O_2_-H_2_O]^−^,	Timosaponin D	Furostanol glycosides	[5, 27]
12	7.49	C_14_H_12_O_5_	259.0607	259.0630	8.88	259 [M-H]^−^, 165 [M-H-C_6_H_6_O]^−^, 121 [M-H-C_6_H_6_O-CO_2_]^−^,	2,6,4′-Trihydroxy-4-methoxyBenzophenone	Benzophenones	[26]
13	8.12	C_56_H_92_O_28_	1211.5697	1211.5642	4.54	1211 [M-H]^−^, 1193 [M-H-H_2_O]^−^, 271 [M-H-H_2_O-C_5_H_8_O_4_-4C_6_H_10_O_5_-C_8_H_14_O_2_]^−^, 253 [M-H-2H_2_O-C_5_H_8_O_4_-4C_6_H_10_O_5_-C_8_H_14_O_2_]^−^,	Timosaponin H1	Furostanol glycosides	[5, 27]
14	8.26	C_56_H_94_O_28_	1213.5854	1213.5803	4.20	1213 [M-H]^−^, 273 [M-H-H_2_O-C_5_H_8_O_4_-4C_6_H_10_O_5_-C_8_H_14_O_2_]^−^, 255 [M-H-2H_2_O-C_5_H_8_O_4_-4C_6_H_10_O_5_-C_8_H_14_O_2_]^−^	Timosaponin I1/D1	Furostanol glycosides	[5, 27]
15	8.48	C_45_H_76_O_19_	919.4903	919.4882	2.28	919 [M-H]^−^, 901 [M-H-H_2_O]^−^, 301 [M-H-H_2_O-3C_6_H_10_O_5_-C_6_H_10_O_2_]^−^, 273 [M-H-H_2_O-3C_6_H_10_O_5_-C_8_H_14_O_2_]^−^, 255 [M-H-2H_2_O-3C_6_H_10_O_5_-C_8_H_14_O_2_]^−^,	Timosaponin B II or Timosaponin L or (25S)-Officinalisnin-I	Furostanol glycosides	[5, 27]
16	9.60	C_39_H_64_O_15_	771.4167	771.4174	0.91	771 [M-H]^−^, 609 [M-H-C_6_H_10_O_5_]^−^, 289 [M-H-2C_6_H_10_O_5_-C_8_H_14_O_3_]^−^, 271 [M-H-2C_6_H_10_O_5_-C_8_H_14_O_3_-H_2_O]^−^, 253 [M-H-2C_6_H_10_O_5_-C_8_H_14_O_3_-2H_2_O]^−^,	Timosaponin F (C39)	Spirostanol glycosides	[27]
17	10.01	C_45_H_74_O_18_	901.4797	901.4773	2.66	901 [M-H]^−^, 739 [M-H-C_6_H_10_O_5_]^−^, 577 [M-H-2C_6_H_10_O_5_]^−^, 273 [M-H-3C_6_H_10_O_5_-C_8_H_14_O_2_]^−^, 255 [M-H-3C_6_H_10_O_5_-C_8_H_14_O_2_-H_2_O]^−^,	Xilingsaponin B	Spirostanol glycosides	[27]
18	10.14	C_45_H_74_O_18_	901.4797	901.4768	3.22	739 [M-H-C_6_H_10_O_5_]^−^, 577 [M-H-2C_6_H_10_O_5_]^−^, 273 [M-H-3C_6_H_10_O_5_-C_8_H_14_O_2_]^−^, 255 [M-H-3C_6_H_10_O_5_-C_8_H_14_O_2_-H_2_O]^−^,	Timosaponin B III	Furostanol glycosides	[5, 27]
19	10.23	C_45_H_74_O_18_	901.4797	901.4807	1.11	739 [M-H-C_6_H_10_O_5_]^−^, 577 [M-H-2C_6_H_10_O_5_]^−^, 273 [M-H-3C_6_H_10_O_5_-C_8_H_14_O_2_]^−^, 255 [M-H-3C_6_H_10_O_5_-C_8_H_14_O_2_-H_2_O]^−^,	Macrostemonoside F or Timosaponin C	Furostanol glycosides	[5, 27]
20	10.28	C_51_H_84_O_23_	1063.5325	1063.5352	2.54	1063 [M-H]^−^, 273 [M-H-4C_6_H_10_O_5_-C_8_H_14_O_2_]^−^, 255 [M-H-4C_6_H_10_O_5_-C_8_H_14_O_2_-H_2_O]^−^,	Timosaponin B IV (C51)	Furostanol glycosides	[5, 27]
21	10.60	C_45_H_74_O_18_	901.4797	901.4824	3.00	739 [M-H-C_6_H_10_O_5_]^−^, 577 [M-H-2C_6_H_10_O_5_]^−^, 273 [M-H-3C_6_H_10_O_5_-C_8_H_14_O_2_]^−^, 255 [M-H-3C_6_H_10_O_5_-C_8_H_14_O_2_-H_2_O]^−^,	Macrostemonoside F or Timosaponin C	Furostanol glycosides	[5, 27]
22	10.69	C_15_H_12_O_4_	255.0658	255.0666	3.14	255 [M-H]^−^, 135 [M-H-C_7_H_4_O_2_]^−^, 119 [M-H-C_7_H_4_O_3_]^−^,	2′,4′,4-Trihydroxychalcone	Chalcone-type flavonoids	[29]
23	10.73	C_17_H_16_O_3_	267.1021	267.1036	5.62	267 [M-H]^−^, 119 [M-H-C_9_H_8_O_2_]^−^, 107 [M-H-C_10_H_8_O_2_]^−^, 91 [M-H-C_10_H_8_O_3_]^−^,	Oxy- hinokiresinol	Coumarins	[5]
24	11.00	C_16_H_14_O_5_	285.0763	285.0777	4.91	285 [M-H]^−^, 165 [M-H-C_7_H_4_O_2_]^−^, 119 [M-H-C_8_H_6_O_4_]^−^,	(2S)-7,4′-Dihydroxy-5-methoxyflavone	Flavanones	[25]
25	11.23	C_16_H_16_O_5_	287.0920	287.0931	3.83	287 [M-H]^−^, 269 [M-H-H_2_O]^−^, 259 [M-H-CO]^−^,	2′-O-Methylphloretin	Chalcone-type flavonoids	[25]
26	11.38	C_39_H_64_O_14_	755.4218	755.4218	0.00	755 [M-H]^−^, 593 [M-H-C_6_H_10_O_5_]^−^, 575 [M-H-H_2_O-C_6_H_10_O_5_]^−^, 431 [M-H-2C_6_H_10_O_5_]^−^, 271 [M-H-2C_6_H_10_O_5_-C_8_H_14_O_2_-H_2_O]^−^, 253 [M-H-2C_6_H_10_O_5_-C_8_H_14_O_2_-2H_2_O]^−^,	Timosaponin A II or Timosaponin G (C39) or *Anemarrhena* sapinin III	Spirostanol glycosides	[5, 27]
27	12.25	C_16_H_18_O_3_	257.1178	257.1190	4.67	257 [M-H]^−^, 107 [M-H-C_9_H_10_O_2_]^−^, 91 [M-H-C_9_H_10_O_3_]^−^,	Broussonin A or Broussonin B	Coumarins	[29]
28	13.16	C_17_H_16_O_2_	251.1072	251.1075	1.19	251 [M-H]^−^, 107 [M-H-C_10_H_8_O]^−^, 91 [M-H-C_10_H_8_O_2_]^−^,	Hinokiresinol	Coumarins	[29]
29	16.17	C_39_H_64_O_13_	739.4269	739.4252	2.30	739 [M-H]^−^, 577 [M-H-C_6_H_10_O_5_]^−^, 415 [M-H-2C_6_H_10_O_5_]^−^, 273 [M-H-2C_6_H_10_O_5_-C_8_H_14_O_2_]^−^, 255 [M-H-2C_6_H_10_O_5_-C_8_H_14_O_2_-H_2_O]^−^,	Timosaponin AIII	Spirostanol glycosides	[5, 27]
30	16.25	C_39_H_64_O_13_	739.4269	739.4280	1.49	739 [M-H]^−^, 577 [M-H-C_6_H_10_O_5_]^−^, 415 [M-H-2C_6_H_10_O_5_]^−^, 273 [M-H-2C_6_H_10_O_5_-C_8_H_14_O_2_]^−^, 255 [M-H-2C_6_H_10_O_5_-C_8_H_14_O_2_-H_2_O]^−^,	Timosaponin AIV	Spirostanol glycosides	[5, 27]
31	16.38	C_39_H_64_O_13_	739.4269	739.4286	2.30	739 [M-H]^−^, 577 [M-H-C_6_H_10_O_5_]^−^, 415 [M-H-2C_6_H_10_O_5_]^−^, 273 [M-H-2C_6_H_10_O_5_-C_8_H_14_O_2_]^−^, 255 [M-H-2C_6_H_10_O_5_-C_8_H_14_O_2_-H_2_O]^−^,	Smilageninoside	Spirostanol glycosides	[5, 27]
32	16.73	C_18_H_18_O_2_	265.1229	265.1235	2.26	265 [M-H]^−^, 121 [M-H-C_10_H_8_O]^−^,	Monomethyl-cis-hinokiresinol	Coumarins	[29]
